# Impact of aerobic exercises on taste perception for sucrose in patients with type 2 diabetes mellitus; A randomized controlled trial

**DOI:** 10.1186/s12902-022-00936-5

**Published:** 2022-01-15

**Authors:** Dinithi Vidanage, Shamini Prathapan, Priyadarshika Hettiarachchi, Sudharshani Wasalathanthri

**Affiliations:** 1 Department of Nursing & Midwifery, Faculty of Allied Health Sciences, General Sir John Kotelawala Defence University, Kandawala Road, Ratmalana, 10390 Sri Lanka; 2 Department of Community Medicine, Faculty of Medical Sciences, University of Sri Jayewardenepura, Gangodawila, Nugegoda, 10250 Sri Lanka; 3 Department of Physiology, Faculty of Medical Sciences, University of Sri Jayewardenepura, Gangodawila, Nugegoda, 10250 Sri Lanka; 4 Department of Physiology, Faculty of Medicine, University of Colombo, Kinsey Road, Colombo 08, 00800 Sri Lanka

**Keywords:** Aerobic exercises, Taste perception, Type 2 diabetes mellitus

## Abstract

**Background:**

Regular exercise is a key element in the management of type 2 diabetes mellitus (T2DM). Although the importance of regular exercises on glycemic control in people with diabetes is studied extensively, evidence is lacking on its impact on sweet taste perception. Thus, the aim of this study was to determine the impact of aerobic exercises on taste perception for sucrose in people with diabetes.

**Methods:**

A sample of 225 people with diabetes aged 35-60 years was assigned randomly into 3 groups; aerobic exercise, combined exercise and a control group. The outcomes of the combined exercise group is not reported. The aerobic exercise group performed brisk walking 30min/day, 4-5days/week for 6 months. The primary outcome measures were supra-threshold intensity ratings and preference for sucrose assessed at baseline, at 3 and 6 months using ‘general Labeled Magnitude Scale’ and ‘Monell 2-series-forced choice method’ respectively. Glycated haemoglobin (HbA1c) level was assessed at baseline and at 6 months to determine glycemic control.

**Results:**

Aerobic exercise group showed significantly increased ratings (mm) for higher sucrose concentrations at 3 months (mean difference for 2.02M; +6.63±2.50, *p*=0.048 and for 0.64M; +7.26±2.76, *p*=0.026) and at 6 months (mean difference for 0.64M; +7.79±4.49, *p*= 0.044) compared to baseline and also when compared to controls (mean difference for 2.02M between baseline and 3 months; intervention: +6.63±2.50, control: -4.01±1.79, *p*=0.02 and between baseline and 6 months for 2.02M; intervention: +3.15±0.57, control: -7.96±0.40, *p*=0.022 and for 0.64M; intervention: +7.79±4.49, control: -8.98±0.99, *p*=0.003). A significantly reduced preference (mol/L) was seen both at 3 (mean difference; -0.03±0.02, *p*= 0.037) and at 6 months (mean difference; -0.05±0.12, *p*=0.011) compared to baseline within the intervention group. Also, a significant reduction was seen in the intervention group compared to controls at 6 months (mean difference; intervention: -0.05±0.12, control: 0.01±0.03, *p*=0.044). HbA1c was significantly reduced in the intervention group compared to controls at 6 months (mean difference; intervention -0.43±1.6%, control +0.33±1.8%, *p*=0.018).

**Conclusion:**

Regular aerobic exercises increase the sweet taste sensitivity, especially for higher concentrations of sucrose and decrease sweet taste preference in people with diabetes . These alterations in sweet taste perception, are likely to contribute to a better glycemic control in people with diabetes.

**Trial registration:**

This trial was registered at the Sri Lanka Clinical Trial registry on 16/12/2015. (Trial registration number- SLCTR/2015/029, https://slctr.lk/trials/slctr-2015-029).

## Background

The prevalence of type 2 diabetes mellitus (T2DM) has escalated to epidemic proportions across the globe in developing as well as in developed countries over the past few decades [1–3]. The number of people with diabetes continues to rise globally and is expected to be increased by 55% in 2035 [4]. Healthy food practices and regular physical activity are fundamental aspects of lifestyle habits in diabetic care [5]. Poor dietary habits are associated with poor glycemic control [2] and the current diabetic guidelines emphasize limiting the intake of sugar, sugar-sweetened beverages and other food items to optimize the glycemic profile. Further, it is recommended to consume <10% of calories per day from added sugars as per the calorie limits for healthy eating practices in people with diabetes [6]. In addition, regular physical exercises are considered a first-line intervention for the prevention and treatment of T2DM as exercises are proven to increase insulin sensitivity [7].

Taste sensitivity plays a vital role when determining food choices. The supra-threshold intensity perception range refers to the perceived sweetness above the recognition threshold, which is defined as the lowest concentration at which the sweet taste quality can be identified [8–10]. Beyond this point, the perceived sweetness ranges from just perceivable to strong, until it reaches the individual’s terminal threshold for sucrose, beyond which any increase in concentration no longer causes consequential increase in perceived sweetness intensity [11].

Moreover, the perceived intensity ratings for supra-threshold concentrations of sucrose are reported to be lower in people with diabetes when compared to non-diabetic individuals implying the necessity of higher quantities of sugar to perceive the same sweet taste [12, 13] driving them to over-consume sugar which may result in high blood glucose concentrations [14]. Further, the sweet taste produced by sucrose and other sugars is shown to modulate the eating behavior through the activation of neurotransmitters such as dopamine and endogenous opioids in the brain reward system [15].

Previous research has reported an association between physical exercise and taste perception. Aerobic exercises for 4 and 8 weeks were shown to increase the sweet taste sensitivity in healthy individuals [16]. In another study, young female swimmers were shown to perceive a sucrose solution as sweeter when compared to less active counterparts [17], Further, a recent study has reported significantly higher intensity ratings (perceived) for sucrose in active men performing more than 4 structured exercise sessions per week for 6 months compared to inactive men [18]. When considering the effect of exercises on preference for sweet taste, previous reports show a higher preference for sucrose after exercises [17, 19] in non-diabetic individuals.

Although exercises seem to play a vital role in modulating taste sensitivity, limited data are available assessing this relationship in people with diabetes despite energy homeostasis being a prime attribute in their glycemic control. If these interrelated factors are proven to be altered via regular exercises, it would be promising for better blood glucose levels in people with diabetes . Since there is a dearth of literature that has critically investigated all these factors together, the present prospective study was aimed at determining the impact of long-term aerobic exercises on taste perception for sucrose in people with diabetes.

## Methods

### Trial design and setting

This is a randomized controlled trial (parallel-group trial design with allocation ratio 1:1) conducted at the Department of Physiology, University of Sri Jayewardenepura, Sri Lanka. This trial was first registered at the Sri Lanka Clinical Trial Registry on 16/12/2015, registration number - SLCTR/2015/029.

### Sample size

A randomized controlled trial sample size formula with type one (α) and type two (β) errors of 0.05 and the power of 80% was used to calculate the sample size. Mean values of HbA1c % in a previously published randomized controlled trial were used as the key variable for calculation [20]. According to the calculation, 75 people with diabetes were enrolled in each group, assuming a drop out of 11%.

### Participants

People with diabetes aged 35–60 years with a history of T2DM for more than 5 years, with HbA1c between 6.6% - 9.9% and were willing to perform regular exercises were included in the study. Those with diseases of the oral cavity, smokers, betel chewers and regular alcohol consumers were excluded as these affect taste sensitivity [21]. Participants with ischemic heart disease, uncontrolled hypertension (systolic blood pressure > 160 mmHg), psychiatric illnesses and, physical and neurological disorders that affect exercises were also excluded from the study. Since medications affect taste perception [22], participants were advised to report any change of their medications immediately.

Those who were on insulin were excluded since it is reported that insulin affects taste sensitivity [23].

### Selection of patients

A systematic random sampling of people with diabetes conforming to the criteria under the study population was selected from the Family Practice Centre, of the University of Sri Jayewardenepura from October 2016 to January 2018. The skip interval was taken as 10, and thus every 10^th^ participant who met the criteria was selected into the study. People with diabetes were enrolled in the study after taking the consent once they were found to be eligible. A serial number was assigned in chronological order for data entering purposes. A sample of 225 people with diabetes was assigned into 3 groups (aerobic exercise group, combined exercise group and a control group) in on-going basis using simple randomization (parallel design, allocation ratio 1:1). The allocation was done using a random number table. An arbitrary point was chosen from the random number table, which is a 3-digit number (since the total sample size carries a 3-digit number). The randomly picked number was matched with the participants’ serial numbers. Thus, the 1^st^ matched number was assigned to the aerobic group, the 2^nd^ matched number to the combined group and the 3^rd^ number to the control group. Enrolling people with diabetes and assigning them to the 3 groups were done by the principal investigator. The assignment information was kept under lock and key. The treating physicians did not involve in data collection but supervised the study as a whole. The code was broken in case of any unwanted severe adverse effect occurred in a participant and it was done by the treating physicians who were members of the study team. None of the participants were made aware of the randomization at any given point of time. However, blinding was not possible due to the nature of the intervention.

Each participant was followed up for a period of 6 months. The 2 groups were matched for age, gender, BMI and HbA1c to minimize confounders. The accuracy of study instruments was checked by a pilot study conducted on 10 participants who fulfilled similar inclusion and exclusion criteria. A resting ECG was performed on the participants of the intervention group to assess the cardiovascular fitness for exercise.

### Outcomes

The primary outcome measure was the change of taste perception for sucrose (i.e., supra-threshold intensity ratings and preference) from baseline to 3 and 6 months in the same individual as well as between the aerobic and control groups. The secondary outcome was glycemic control (HbA1c) at 6 months measured by high-performance liquid chromatography.

### Data collection

Baseline characteristics of the participants were recorded at recruitment. Anthropometric measurements and the, taste perception (which includes supra-threshold intensity ratings and preference for sucrose) were assessed at baseline, at 3 and at 6 months in all 3 groups. The glycemic control was assessed at baseline and at 6 months. Data were collected in batches of 5-6 participants per day. On the day of the tests, the participants arrived at the taste laboratory between 08.00 and 8.30 am. They were instructed to abstain from food and beverages from 10 pm the previous day and have 6-8 hours of sleep. Only morning tea/coffee was allowed. On arrival at the study setting, the participants were served with a standard breakfast comprising of a plantain, 3 slices of brown bread with margarine, followed by a glass of water. Anthropometric measurements were taken and 5 ml of blood was drawn under strict aseptic measures for the determination of HbA1_C_ levels. The intervention carried out by the combined group will not be discussed in this manuscript.

### Aerobic exercise intervention

A graded home-based exercise protocol [24] was introduced to the participants in the aerobic group. They were instructed to perform brisk walking for a minimum of 150 minutes per week spread over 4 -5 days (30 minutes/day). To ensure accurate assessment of the exercises, each participant was provided with a ‘pedometer’ that indicates the step count, the distance walked and the number of calories burnt. Participants were instructed to wear the pedometer during exercises and note down the readings after each exercise session. The adherence to the exercise protocol was ensured by telephone reminders in the 2^nd^ week of each month.

### Assessment of taste perception

Taste thresholds were assessed by using general Labeled Magnitude Scale (gLMS) [25]. Concentrations of sucrose solutions used for the estimation of supra-threshold intensity ratings were prepared based on 1/2 log steps (3.16-fold / 10 ^0.25^). The mean recognition threshold obtained in a previous study [13] was used as the barely detectable level. Six cups (5ml each) of sucrose solutions (2.02M, 0.64M, 0.2M, 0.064M, 0.0202M, 0.0064M) in 1-minute gap were presented to each participant in a random order. Participants were asked to taste each sample for 5 s before spitting it out and rate the perceived intensity on a gLMS paper scale. They were instructed to rinse the mouth once with distilled water between each solution. The procedure was repeated 3 times and the average was taken for each person. To train the use of gLMS and to control idiosyncratic scale usage, participants were asked to rate the heaviness of 6 visually identical weights [26].

Taste preference was tested by Monel 2-series forced-choice method. Pairs of sucrose solutions, 5 ml each were presented to the participants. The concentrations of sucrose tested were 0.09M, 0.18M, 0.36M, 0.72M and 1.08M [27]. The procedure was conducted in 2 series. In series 1, concentrations of the middle range were presented (avoiding the highest and the lowest concentrations) in pairs, with the weaker concentration given first. The participants were allowed to taste each solution in the pair for 5 s before choosing the solution they liked. They were instructed to rinse the mouth once with distilled water between the 2 solutions in a pair and rinse twice between each pair. This procedure was continued with varying pairs of different concentrations until the participant chose the solution with the same concentration twice consecutively, which was considered as the preferred solution in series 1. In series 2 which commenced in a gap of 3 minutes, the stronger concentration of the pair was presented first and the same procedure was repeated. The geometric mean of the 2 sucrose concentrations chosen in series 1 and 2 was considered as the preferred concentration of sucrose for a particular participant.

### Statistical analysis

All data were analyzed using SPSS software version 23.0. Normality was checked with Shapiro-Wilk test. Descriptive data of participants were reported as numbers and percentages or means and standard deviations (±SDs). Ratings obtained for taste perception for sucrose at baseline were compared with ratings obtained from the same participant at 3 and at 6 months by paired sample t-test in the intervention and control group separately. In addition, mean differences obtained between 0-3 months and 0-6 months in the aerobic group were compared with that of the control group by independent sample t- test. Mixed between-within subject ANOVA (repeated measures), [i.e., time (within-subject factor) and group (between-subject factor)] was performed to assess the impact of 2 conditions (aerobic /control) on taste perception for sucrose (supra-threshold intensity ratings and preference) across 3 time points (i.e., baseline, 3 months and 6 months).

HbA1c values between the baseline and 6 months were compared in the intervention group by the paired sample t-test. Mean differences in HbA1c between 0-6 months in the aerobic group were compared with those of the control group by the independent sample t-test. The impact of the intervention on HbA1c levels was assessed by mixed between-within subject ANOVA (repeated measures) [i.e., time (within-subject factor) and group (between-subject factor)].

### Managing missing data

There were no missing data at the baseline. Therefore, baseline data of all participants were analyzed to show socio-demographic, clinical and anthropometric findings at the recruitment. However, there were missing data at 3 and 6 months due to the dropouts. Thus, all participants with missing data in both intervention and control groups were removed from the datasheet and not considered for analysis of primary and secondary outcomes. Thus, data of 20 participants (13%) at 3 months and of additional 12 participants (8%) at 6 months were removed from the data sheet.

### Ethical considerations

The study was performed in accordance with the guidelines and regulations of the Helsinki declaration and the protocol was approved by the Ethics Review Committee (ERC approval no. 23/15) of the Faculty of Medical Sciences, University of Sri Jayewardenepura, Sri Lanka. Informed written consent was obtained from each participant prior to data collection.

## Results

**Fig. 1:**
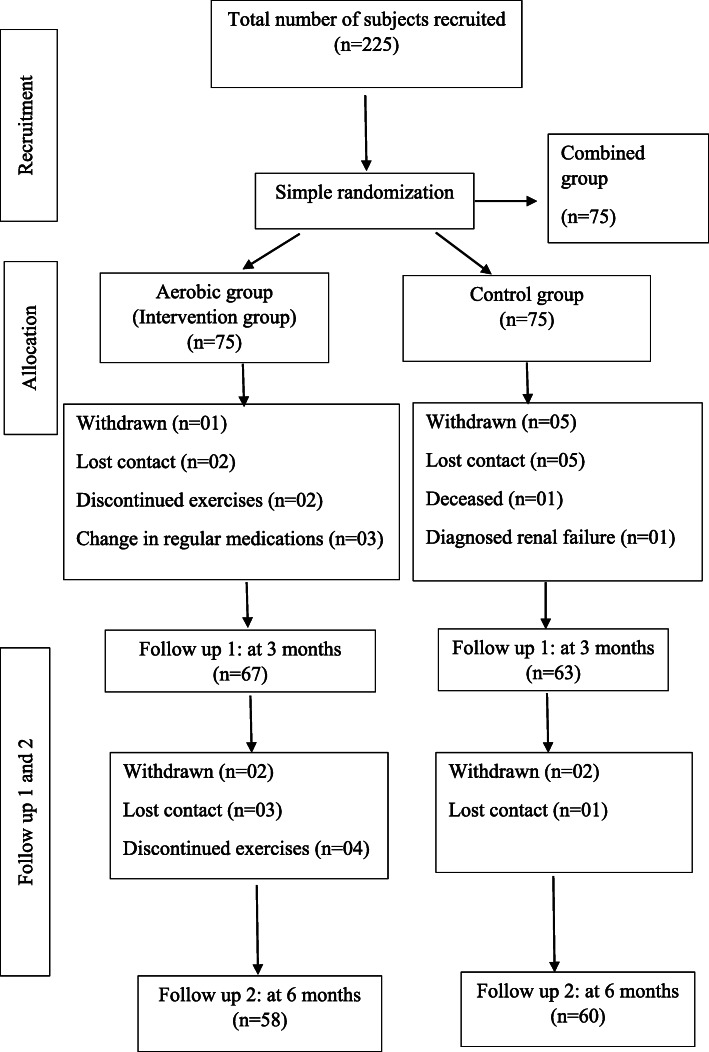
Flow diagram showing the outcome of the aerobic exercise group and the control group as per the CONSORT (2010) guidelines

Sixty-seven participants in the aerobic group and 63 participants in the control group completed the study with a response rate of 87% (*n*=130) at 3 months. Seventy nine percent of the participants (*n*=118) remained until completion of the study at 6 months (i.e., intervention = 58, control = 60). None of the participants reported any adverse effects due to the intervention. Figure 1 shows the outcome of the recruited subjects.

Results of the combined exercise group are not presented in this manuscript . Therefore, the aerobic exercise group is referred to as the intervention group.

Baseline socio-demographic, anthropometric and clinical characteristics of participants in the intervention and control groups are shown in Table 1. The mean age ±SD of the intervention and control groups were 53.20±6.38 years and 54.70±5.41 years respectively. A little more than half of the cohort of participants were females (i.e., 56% in the intervention group and 57.4% in the control group). Both groups reported a history of T2DM for more than 8 years. Metformin was the commonly used oral hypoglycemic agent by participants in both groups. The intervention group reported a mean HbA1c ±SD of 8.07±1.67 %, while it was 7.94±1.14 % in the control group.

**Table 1:** Baseline characteristics of study participants

Variable	Aerobic exercise group (*n*=75)	Control group (*n*=75)	*p*-value
Age (years) ^a^	53.20±6.38	54.70±5.41	0.112
Gender, n (%)			
Male	33 (44%)	32 (42.6%)	0.500
Female	42 (56%)	43 (57.4%)	
Education level, n (%)			
No education	0	0	0.085
Grade 1 – 5	0	01 (1.3%)	
Grade 6-10	20 (26.7%)	16 (21.3%)	
Up to Ordinary level	27 (36%)	39 (52%)	
Up to Advanced Level	19 (25.3%)	17 (22.7%)	
Tertiary education	09 (12%)	02 (2.7%)	
Civil status, n (%)			
Married	72 (96%)	66 (88%)	-
Unmarried	03 (4%)	09 (12%)	
Monthly income/person Sri Lankan rupees (LKR), n (%)			
No personal income	03 (4%)	22 (29.4%)	-
<10,000	02 (2.7%)	06 (8%)	
between 10,000-20,000	17 (22.6%)	12 (16%)	
> 20,000	53 (70.7%)	35 (46.6%)	
Duration of T2DM (years) ^a^	8.56±4.92	9.60±6.35	0.265
Mode of treatment, n (%)			
On their own	07 (9.3%)	13 (17.3%)	-
Regular clinic visits	28 (37.3%)	49 (65.4%)	
Only when required	40 (53.4%)	13 (17.3%)	
Use of oral hypoglycemic agents, n (%)			
Not used	06 (8%)	07 (9.3%)	0.174
Metformin	64 (85.3%)	56 (74.7%)	
Other hypoglycemics	05 (6.7%)	12 (16%)	
Frequency of checking fasting blood glucose level (FBG), n (%)			-
Once a month	43 (57.3%)	49 (65.3%)	
Once in 2-3 months	31 (41.4%)	25 (33.4%)	
No regular checking of FBG	01 (1.3%)	01 (1.3%)	
Family History of T2DM, n (%)			-
Yes	49 (65.3%)	43 (57.3%)	
No	26 (34.7%)	32 (42.7%)	
Body mass index (BMI) (kg/m^2^) ^a^	24.60±3.70	25.28±3.03	0.220
HbA1c (%) ^a^	8.07±1.67	7.94±1.14	0.578

^a^Values are expressed as mean ± SD

### The impact of exercises on taste perception for sucrose

**Fig. 2:**
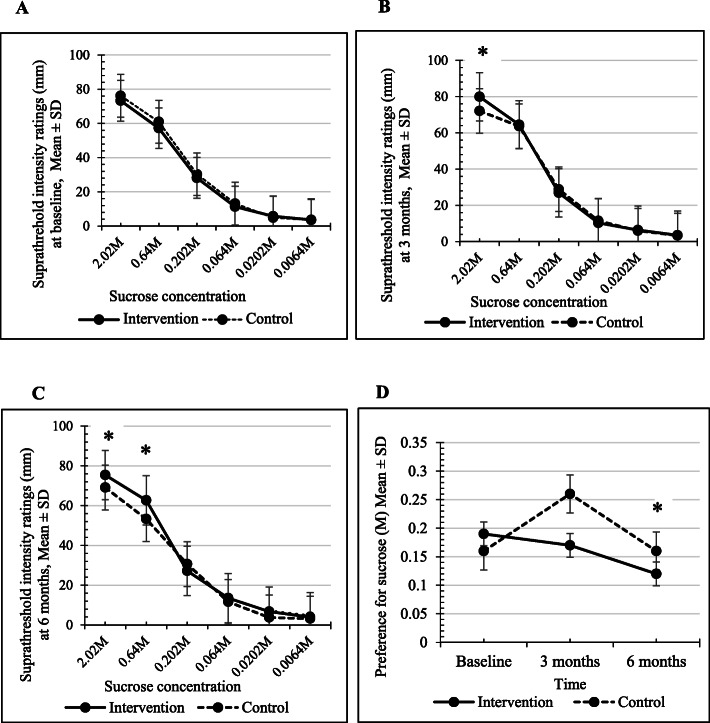
Supra-threshold intensity ratings in the intervention group and the control group at baseline (A), at 3 months (B) and 6 months (C) are shown. At 3 months, the intervention group showed a significantly higher rating compared to controls for the highest concentration (i.e.,2.02M). At 6 months, the intervention group showed a significantly higher rating compared to controls for the highest (i.e.,2.02M) and the 2^nd^ highest concentration of sucrose (i.e.,0.64M). The preference for sucrose in the intervention group was significantly lower compared to the controls at 6 months (D). All data are expressed as mean ± SD. *p <0.05

At baseline, there was no significant differences in the supra-threshold intensity ratings for sucrose between the intervention and the control group (Fig. 2A). Participants in the intervention group showed increased supra-threshold intensity ratings for 3 out of 6 sucrose solutions, with the results being statistically significant for the highest (i.e.,2.02M) and the 2^nd^ highest (i.e.,0.64M) concentrations compared to the baseline at 3 months (Table 2). In the control group, none of the solutions showed statistically significant differences in supra-threshold intensity ratings between baseline and 3 months (Data not shown). At 3 months, the intervention group showed a significantly higher rating compared to controls for the highest concentration (i.e.,2.02M) (Fig. 2B).

**Table 2:** Comparison of supra-threshold intensity ratings for sucrose at 3 and 6 months with the baseline value in the intervention group

Sucrose concentration mol/L (M)	Supra-threshold intensity ratings (mm)					
	(Mean ± SD) (*n*=67)		*p* value	(Mean ± SD) (*n*=58)		*p* value
	at baseline	at 3 months		at baseline	at 6 months	
2.02M	73.28±21.73	79.92±16.53	0.048^a^	72.28±21.97	75.43±22.54	0.295
0.64M	57.23±23.41	64.50±16.44	0.026^a^	54.87±22.63	62.66±27.12	0.044^a^
0.202M	28.14±16.35	26.93±10.27	0.580	26.26±16.35	27.20±14.20	0.660
0.064M	11.33±11.76	10.33±9.38	0.567	11.35±12.31	13.52±10.24	0.274
0.0202M	5.75±8.20	6.39±6.10	0.607	5.81±8.67	6.71±6.66	0.473
0.0064M	3.60±5.83	3.52±4.78	0.929	3.76±6.17	3.94±4.42	0.841

^a^Level of significance < 0.05, values are expressed as mean ± SD (paired sample t-test)

When the mean differences were compared at 3 months, the intervention group showed a significantly higher rating compared to controls for the highest concentration of sucrose studied (Table 3).

**Table 3:** Comparison of supra-threshold intensity ratings between intervention and control group

Sucrose concentration mol/L (M)	Mean differences ± SD between 0 and 3 months (mm)			Mean differences ± SD between 0 and 6 months (mm)		
	Intervention (*n*=67)	Control (*n*=63)	*p* value	Intervention (*n*=58)	Control (*n*=60)	*p* value
2.02M	+6.63±2.50	-4.01±1.79	0.020^a^	+3.15±0.57	-7.96±0.40	0.022^a^
0.64M	+7.26±2.76	+2.72±8.68	0.276	+7.79±4.49	-8.98±0.99	0.003^a^
0.202M	-1.20±1.94	-1.44±4.15	0.947	+0.93±1.59	-0.16±5.01	0.815
0.064M	-1.00±2.15	-1.66±5.26	0.861	+2.16±2.07	-1.69±4.28	0.148
0.0202M	+0.64±1.47	+1.14±0.18	0.904	+0.89±2.01	-1.20±2.57	0.141
0.0064M	-0.08±1.02	-0.16±1.27	0.885	+0.17±1.75	-0.34±2.46	0.633

^a^Level of significance *p*<0.05, values are expressed as mean ± SD (Independent sample t-test)

At 6 months, the supra-threshold intensity ratings of all sucrose solutions showed an increase in the intervention group with a significant increase for the 2^nd^ -highest concentration in comparison with the baseline values (Table 2). In contrast in the control group, supra-threshold intensity ratings were not increased in any of the concentrations and significant reductions were evident in the highest and the 2^nd^ -highest concentrations (Data not shown). At 6 months, the intervention group showed a significantly higher rating compared to controls for the highest (i.e.,2.02M) and the 2^nd^ highest concentration of sucrose (i.e.,0.64M) (Fig. 2C).

When the mean differences were compared at 6 months, the supra-threshold intensity ratings were significantly increased for 2.02M and 0.64M concentrations in the intervention group compared to controls indicating a significant increase in the sweet taste sensitivity for higher sucrose concentrations in physically active people with diabetes compared to their inactive counterparts (Table 3).

When the ANOVA results were considered, a significant main effect of time was not observed [F (2, 115) = 1.23, *p*= 0.295, partial eta squared=0.021] but a significant effect was observed for time x group interaction [F (2, 115) = 3.87, *p*=0.024, partial eta squared = 0.063] for the supra-threshold intensity ratings of the highest sucrose solution (2.02M) across the 3 time points. However, the main effect comparing the 2 groups was not statistically significant [F (1, 116) = 1.08, *p*= 0.299, partial eta squared = 0.009].

In the 2^nd^ highest concentration (0.64M), a significant main effect of time [F (2, 115) = 4.13, *p*=0.018, partial eta squared=0.067] and time x group interaction [F (2,115) = 4.57, *p*=0.012, partial eta squared = 0.074] was observed, with no significant effect between groups [F (1,116) = 0.02, *p*=0.889, partial eta squared =. 0.00].

At baseline, there were no significant difference in the preference for sucrose between the intervention and the control group (Fig. 2D). However, the preference was significantly reduced in the intervention group at 6 months compared to controls (Fig. 2D). The preference for sucrose was significantly reduced following aerobic exercises for 3 (intervention group, baseline; 0.19± 0.17, 3 months; 0.15±0.15, t (66) =2.13, *p*=0.037) and for 6 months (baseline; 0.17±0.16, 6 months; 0.12±0.04, t (57) =2.61, *p*=0.011) in the same individual. In the control group, a significant change was not observed in the individuals at 3 (baseline; 0.16±0.08, 3 months; 0.26±0.1, t (62) = -0.71, *p*=0.479) and at 6 months (baseline; 0.16±0.08, 6 months; 0.16±0.10, t (59) = 0.04, *p*= 0.968) compared to the baseline (Fig. 2D).

 When the mean differences for sucrose preference were compared between the 2 groups, a significant reduction was observed only at 6 months (intervention: -0.05±0.12, control: 0.01±0.03, t (116) =-2.03, *p*=0.044) in the intervention group compared to controls (Table 4).

**Table 4:** Comparison of taste preference for sucrose between intervention and control group

Mean differences ± SD between 0 and 3 months (mol/L)			Mean differences ± SD between 0 and 6 months (mol/L)		
Intervention (*n*=67)	Control (*n*=63)	*p* value	Intervention (*n*=58)	Control (*n*=60)	*p* value
-0.03±0.02	+0.09±1.04	0.309	-0.05±0.12	-0.01±0.03	0.044^a^

^a^Level of significance p<0.05, values are expressed as mean ± SD (Independent sample t-test)

Repeated measures ANOVA revealed that there was a significant effect of time [F (2, 115) = 4.66, *p*=0.011, partial eta squared = 0.075] and time x group interaction [F (2, 115) = 3.68, *p*=0.028, partial eta squared = 0.06] on preference for sucrose over the 3 time points. However, the main effect comparing the 2 groups was not statistically significant [F (1, 116) = 0.023, *p*=0.879, partial eta squared= 0.00].

### The impact of exercises on glycemic control

The intervention group showed significantly reduced HbA1c levels after 6 months of regular aerobic exercises when compared to baseline (baseline; 8.0±1.67%; at 6 months; 7.6±1.45%; t (57) =2.02, *p*=0.047). When the mean differences between 0-6 months were compared between the 2 groups, the intervention group showed a significant reduction in HbA1c compared to the control group i.e., intervention; -0.43±1.6% control; +0.33±1.8%, t (116) = -2.4, *p*=0.018. Repeated measures ANOVA revealed that there was a significant time x group interaction [F (1,116) = 5.7, *p*=0.018, partial eta squared=0.018] on HbA1c over 6 months.

## Discussion

According to our knowledge, the present study is the only reported investigation assessing the impact of regular aerobic exercises on taste sensitivity in people with diabetes.

When considering the self-care activities of Sri Lankan people with diabetes, it is reported that they are more likely to adhere to dietary control and regular medications than engaging in regular exercises, with only less than 50% engaging in regular physical exercises [28, 29]. In contrast, in Ghana, exercise was the most commonly performed self-care activity in diabetics [30]. In the intervention group of the present prospective study, the introduction of a specific exercise protocol and regular follow-up with frequent telephone reminders are the likely reasons to achieve a 77% (*n*=58) adherence to regular physical exercises. A Japanese study investigating the factors for the continuation of exercises in diabetic patients has also reported that goal setting and consistent instructions on exercises facilitate adherence [31]. In the cross-sectional study conducted by Saumika et al. in 2019 [28], the participants aged more than 50 years were less likely to follow regular exercise regimens compared to their younger counterparts. In addition, another cross-sectional study in Nepal reported that nearly 1/5^th^ of the diabetics in their study were physically inactive, where females were more inactive than males [32]. However, in the current study, the exercise dropouts were significantly lower in the > 50-year age group probably due to the regular and stringent follow-up.

Although medicines are reported to produce taste disturbances [22], it was not possible to exclude participants on hypoglycemics as most people with diabetes depend on them for glycemic control. The majority used metformin and a minority of participants in both intervention and the control groups used other oral hypoglycemic agents namely sulfonylureas e.g., gliclazide and glibenclamide. Since there was no significant difference in the use of oral hypoglycemic agents between the 2 groups (*p*=0.174), the effect of these drugs on taste was assumed to be equal. Those who were on insulin were excluded since it is reported that insulin affects taste sensitivity [23], which is an exclusion criterion in the study**.**

Taste impairment for sucrose and similar tastes is well documented in people with diabetes [13, 33–35]. Although the exact mechanism is yet to be elucidated, neuropathy defects and/or morphological changes of taste buds due to diabetes are postulated by studying diabetic rats [36]. However, studies carried out to assess the effect of exercises on taste perception have been limited to healthy adults [16, 18] and restricted to the assessment of recognition [16, 35] and detection thresholds [37, 38]. In the present study, exercise-induced changes in taste perception were assessed in people with diabetes. Since it is believed that supra-threshold intensity ratings reflect the actual taste world of an individual [9], we have assessed the impact of exercises on intensity ratings of 6 supra-threshold concentrations of sucrose.

Habitual exercise is shown to influence the taste in healthy individuals [18]. In a study comparing the taste perception between active and inactive men, Feeny et al., [18] have shown that active men perceive a greater intensity of sweet taste, especially for high concentrations of sucrose. This finding was supported by another study which showed an increase in taste sensitivity following 8 weeks of aerobic exercises in healthy males [16]. However, the findings on taste thresholds following a single bout of exercise have revealed contradictory findings. Although the taste thresholds were shown to decrease during the period of fatigue after a 2-hour half marathon [37], another study by the same investigators did not show a change in taste sensitivity following rigorous exercises for a more prolonged duration [39].

In the present study, an increase in supra-threshold intensity ratings for sucrose has been observed in response to regular aerobic exercises for durations of 3 and 6 months, particularly for higher concentrations of sucrose. Nakanishi et al. [40] suggested intensity of exercise as an important factor in making an impact on the sensitivity to sweet taste. In his study, a group of healthy young adults who performed high-intensity cycling exercises showed an increased sensitivity to sweetness compared to the ones who performed low-intensity exercises. However, some investigators have shown that sensitivity to sweet taste is related to changes in plasma glucose regardless of the exercise intensity [40], and it is logical to postulate that changes in sweet taste sensitivity is related to the energy demands of an individual. In the present study, the intervention group showed significantly reduced HbA1c values after 6 months of regular aerobic exercises when compared to their own baseline and also to the control group suggesting a better glycemic control achieved through changes in sweet taste perception. However, it is also likely that better glycemic control achieved through regular exercises has contributed to the increased sensitivity for sweet taste. Further studies are needed to explore the exact causal relationship.

With regards to the long-term effects of aerobic exercises, increased regeneration and expression of taste receptor cells may contribute to improved responsiveness of these cells thus leading to increased sweet taste sensitivity [16]. Moreover, some investigators suggest the adipocyte hormone leptin as the link between exercises and increased sweet taste sensitivity as they have observed decreased serum leptin and sweet taste thresholds following weight loss [38]. Long-term exercises are proven to increase the secretion of hormones such as GLP-1 [41] and PYY [42] which are proven to modulate taste sensitivity [43, 44]. Although assessing the changes in these hormone levels was beyond the scope of this study, the alterations in the hormone milieu may be speculated to be the link between the changes in taste sensitivity.

People with diabetes crave for sugar [45] probably because they are less sensitive to sweet taste [12, 33, 46]. In addition, they are known to crave for dietary carbohydrates [47]. Yu and co-researchers also suggest that the carbohydrate craving is associated with poor glycemic control and craving scores decline with improvements in glycemic profile [47]. Since the actual sugar intake correlates with the preference for sugar [45], an intervention which would reduce the preference is expected to reduce the sugar intake leading to better glycemic control.

In the present study, the preference for sucrose in the intervention group showed a significant reduction following 6 months of regular exercises, compared to the control group. Although reported studies investigating the effect of exercises on preference for sugar taste are sparse in people with diabetes, inconsistent results are observed in studies performed on healthy individuals [19, 39, 48]. A recent investigation comparing the taste perception between habitually exercising and inactive men does not report a difference in preference for sweet taste between the 2 groups although they have observed an increase in perceived sweet taste intensity in active men [18]. An increased preference or liking observed for sucrose immediately following a single bout of exercise for a short duration [19, 48] may be attributed to the high demand for metabolic energy subsequent to exercise. However, when the exercises were prolonged for a duration of 12 hours, no change in palatability was observed following the exercise [39] and the authors speculate whether it is an effect of extreme exhaustion on sensory testing. In the current study, we observed that in people with diabetes who continued regular exercises, the preference for sweet taste was reduced parallel to the improved sensitivity, both of which in turn led to achieving a better glycemic control. Although the mechanism of reduction of preference for sweet taste in exercising people with diabetes remained unanswered, it is likely that better glycemic control and increased sensitivity associated with regular exercises are instrumental in reducing the sweet taste preference in these people . Yet, further studies are mandatory to confirm this finding and to explore the underlying mechanisms responsible for it.

The current study is unique as it demonstrates the impact of long-term aerobic exercises on sweet taste sensitivity and preference for sucrose simultaneously in a cohort of people with diabetes. The findings were strengthened by comparing the behavior of these parameters in a group of non-exercising controls. As regular exercises play an integral role in diabetic self-management, the current findings are useful as they unravel the behavior of taste perception in people with diabetes in response to regular exercise.

Although the participants were followed up at every 2^nd^ week of each month to assess the exercise adherence, the clinical and biochemical data were not obtained at all these time points due to logistic and financial constraints. Data on taste perception were collected on 3 occasions i.e., baseline, at 3 and 6 months, while HbA1c levels were assessed only at the baseline and at the end of the study at 6 months.

## Conclusions

Regular aerobic exercises alter the taste perception for sucrose i.e., increase the taste sensitivity especially for high concentrations of sucrose and decrease the sweet taste preference in people with diabetes. In addition to the well-established mechanisms of regular exercise induced glycemic changes of diabetics, alterations in the sweet taste perception are likely to contribute to a better glycemic control in people with diabetes.

## Data Availability

The data used to support the findings of this study could be obtained from the corresponding author upon reasonable request.

## Abbreviations

T2DM: Type 2 diabetes mellitus
FBG: Fasting blood glucose
HbA1c: Glycated haemoglobin A 1c
ECG: Electrocardiogram
gLMS: general Labeled Magnitude Scale
GLP-1: Glucagon like peptide -1
PYY: Gut hormone peptide YY
BMI: Body mass index
SLTCR: Sri Lanka Clinical Trial Registry
ERC: Ethics Review Committee
LKR: Sri Lankan rupees
SPSS: Statistical package for the social sciences
